# New Appliances and Things Medical

**Published:** 1904-08-20

**Authors:** 


### NEW APPLIANCES AND THINGS MEDICAL.

[We shall be glad to receive, at our Office, 28 & 29 Southampton Street, Strand, London, W.C., from the manufacturers, specimens of all new preparations and appliances which may be brought out from time to time.]

### WEST SURREY CENTRAL DAIRY COMPANY'S CREAM.

(Guildford.)

We have received a sample of this well-known cream. It is of a good flavour and forms an excellent substitute for fresh cream. Our experience is that even when opened the cream will keep for a reasonable time—two to three days. The extreme value of cream for nutritive purposes and the difficulty of obtaining an adequate supply of fresh cream renders preparations of this kind of great economic value.

### STONE'S LIME JUICE.

(The Finsbury Distillery Company, Ropemaker Street, E.C.)

We have received samples of Stone's Lime Juice and Stone's Lime Juice Cordial, both of which we find to be admirable preparations of their kind. Diluted with a suitable proportion of water, they make pleasant and wholesome beverages.

